# The value of arthroscopy in the treatment of complex ankle fractures – a protocol of a randomised controlled trial

**DOI:** 10.1186/s12891-016-1063-2

**Published:** 2016-05-12

**Authors:** Mareen Braunstein, Sebastian F. Baumbach, Markus Regauer, Wolfgang Böcker, Hans Polzer

**Affiliations:** Department of Trauma Surgery, Munich University Hospital, Ludwig-Maximilians-University Munich, Nußbaumstr 20, Munich, 80336 Germany

**Keywords:** Ankle fracture, Complex ankle fracture, Arthroscopy, Arthroscopically assisted fracture treatment, Chondral lesion, Randomised controlled trial, Foot and ankle surgery

## Abstract

**Background:**

An anatomical reconstruction of the ankle congruity is the important prerequisite in the operative treatment of acute ankle fractures. Despite anatomic restoration patients regularly suffer from residual symptoms after these fractures. There is growing evidence, that a poor outcome is related to the concomitant traumatic intra-articular pathology. By supplementary ankle arthroscopy anatomic reduction can be confirmed and associated intra-articular injuries can be treated. Nevertheless, the vast majority of complex ankle fractures are managed by open reduction and internal fixation (ORIF) only. Up to now, the effectiveness of arthroscopically assisted fracture treatment (AORIF) has not been conclusively determined. Therefore, a prospective randomised study is needed to sufficiently evaluate the effect of AORIF compared to ORIF in complex ankle fractures.

**Methods/design:**

We perform a randomised controlled trial at Munich University Clinic enrolling patients (18–65 years) with an acute ankle fracture (AO 44 A2, A3, B2, B3, C1 - C3 according to AO classification system). Patients meeting the inclusion criteria are randomised to either intervention group (AORIF, *n* = 37) or comparison group (ORIF, *n* = 37). Exclusion criteria are fractures classified as AO type 44 A1 or B1, pilon or plafond-variant injury or open fractures. Primary outcome is the AOFAS Score (American Orthopaedic Foot and Ankle Society). Secondary outcome parameter are JSSF Score (Japanese Society of Surgery of the Foot), Olerud and Molander Score, Karlsson Score, Tegner Activity Scale, SF-12, radiographic analysis, arthroscopic findings of intra-articular lesions, functional assessments, time to return to work/sports and complications. This study protocol is accordant to the SPIRIT 2013 recommendation. Statistical analysis will be performed using SPSS 22.0 (IBM).

**Discussion:**

The subjective and functional outcome of complex ankle fractures is regularly unsatisfying. As these injuries are very common it is essential to improve the postoperative results. Potentially, arthroscopically assisted fracture treatment can significantly improve the outcome by addressing the intra-articular pathologies. Given the absolute lack of studies comparing AORIF to ORIF in complex ankle fractures, this randomised controlled trail is urgently needed to evaluate the effectiveness of additional arthroscopy.

**Trial registration:**

ClinicalTrials.gov reference: NCT02449096 (Trial registration date: April 7th, 2015).

## Background

Acute ankle fractures are one of the leading pathologies disturbing ankle congruence. These fractures are extremely common with an incidence of 0.1–0.2 % per year [1, 2]. Operative treatment performing open reduction and internal fixation (ORIF) is the standard of care for unstable or dislocated ankle fractures with the main goal of anatomical realignment of the joint and restoration of ankle stability [3, 4]. Nevertheless, even successful anatomical reduction does not automatically lead to favourable clinical outcome. According to several studies, the mid- and long-term outcome following operative treatment of acute ankle fractures is often poor [5, 6]. Residual symptoms following ankle fracture include chronic pain, stiffness, recurrent swelling and ankle instability [5, 7]. There is growing evidence that this poor outcome might mostly be related to occult articular injuries involving cartilage and soft tissues [2, 8, 9]. These intra-articular disorders have been shown to negatively affect the clinical results, but it is difficult to diagnose them by physical examination, standard radiography or CT-scans. Magnetic resonance imaging is the most reliable non-invasive diagnostic technique to identify osteochondral lesions (OCL). Nevertheless, in literature its sensitivity varies from 62 to 95 % [10–13]. In case of acute fractures to the ankle, the sensitivity is further reduced due to the traumatic oedema leading to a misjudgement of the appearance and size of OCLs [14]. In this context, many authors have emphasized the value of ankle arthroscopy [6, 7, 15]. In the last decades, it has become a safe and effective diagnostic and therapeutic procedure and has also been proposed in fracture treatment [16]. In ankle fractures, arthroscopically assisted open reduction and internal fixation (AORIF) allows a confirmation of the anatomic reduction, careful examination of the cartilage, capsular and intra-articular ligaments. If necessary, the intra-articular pathologies can immediately be addressed by removing ruptured ligaments and loose bodies, performing chondroplasty or micro fracturing if necessary [15]. There is no evidence that a supplementary ankle arthroscopy leads to a higher complication rates in ankle fracture treatment [8]. On the other hand, small or superficial chondral lesions might heal spontaneously without arthroscopic treatment. Further, the benefit of supplementary ankle arthroscopy in ankle fracture management has not yet been clearly demonstrated. There are only two randomised controlled trials evaluating the effect of additional ankle arthroscopy in isolated fractures of the distal fibula at the level of the syndesmosis (AO type 44 B1). Both documented a high incidence of intra-articular damage. In one study, patients treated arthroscopically showed significant better outcome results. Nevertheless, the vast majority of ankle fractures are managed by open procedures only [17]. Given the absolute lack of RCTs comparing AORIF to ORIF in complex ankle fractures, it is not possible to give recommendations regarding the use of additional arthroscopy up to now. However, several studies could demonstrate that the incidence of OCLs is higher the more complex the fracture is [2, 9]. Based on these findings, it is comprehensive that the effect of arthroscopy might be even more distinctive in complex ankle fractures. Therefore, we perform the first randomised controlled trial intended to report the short-, midterm- and long-term follow-up of patients who underwent operative treatment of complex ankle fractures – with and without ankle arthroscopy.

## Methods/design

### Study design

We perform a randomised controlled study to evaluate the results of the operative treatment of acute ankle fractures with and without ankle arthroscopy. This study protocol is in accordance with the SPIRIT 2013 recommendations. Patients are randomised to either ORIF (comparison) or AORIF (intervention). Patients are recruited at our hospital (level I trauma centre). PICOS criteria are depicted in detail below.

**P**articipants: Patients (18–65 years) with an acute ankle fracture (AO 44-A2, A3, B2, B3, C1-3)
**I**ntervention: Open reduction and internal fixation with ankle arthroscopy (AORIF)
**C**omparison: Open reduction and internal fixation without ankle arthroscopy (ORIF)
**O**utcomes: AOFAS (American Orthopaedic Foot and Ankle Society) Score, JSSF Score (Japanese Society of Surgery of the Foot), Olerud and Molander Score, Karlsson Score, Tegner Activity Scale, SF-12, radiographic analysis, arthroscopic findings of intra-articular lesions, functional assessments, time to return to work/sports, complications
**S**tudy design: Randomised controlled trial

### Patient selection

#### Eligibility criteria

Patients aged 18–65 years with an acute ankle fracture (AO 44 A2, A3, B2, B3, C1-C3) according to the judgment of the surgeons of the foot and ankle team of our level I trauma centre are enrolled in the trial. Fractures are evaluated and graded according to classification reported by AO Foundation. Patients are informed about our current investigation by detailed patient information. Only patients, who confirm in study participation and in the operative procedure, are enrolled. To avoid misclassification, all radiographs are evaluated by at least two orthopaedic surgeons. Disagreements are resolved by discussion. Only patients with a maximum interval of 2 weeks between injury and intervention are included. All patients included must be able to understand the meaning of the trial and its consequences. Written informed consent is mandatory. No additional investigation (clinical or radiographic investigation) will take place if the patient is included compared to patients who refuse inclusion. A list of inclusion and exclusion criteria can be found below (Table 1). Open fractures are excluded. Also, patients with a high risk of anaesthesiology problems (i.e., ASA risk score >3), acute infection, mental illness or low expected compliance are excluded from trial participation. If patients issue a certain treatment preference, they are excluded as well. Patients are further asked for diabetes and smoking as these are possible confounders for the outcome. Moreover, previous ankle injuries are documented in all patients.

**Table 1:** Inclusion and Exclusion criteria

Inclusion criteria	Exclusion criteria
Age 18–65 years	Patients under 18 years or over 65 years
	Patients who have acute infections, mental illnesses, high anesthesiological risk (ASA >3)
	Patients with expected incompliance
Acute ankle fracture classified as AO type 44 A2, A3, B2, B3, C1-C3	Acute ankle fracture classified as AO type 44 A1 or B1 fracture, pilon or plafond-variant injury. Open fractures
Written informed consent (patient is able to read and understand German language properly)	Patients without written informed consent

#### Patient recruitment and screening

All patients presenting to participating surgeons with an acute ankle fracture are screened for participation in the trial. Such patients are classified as 1) excluded, if they do not meet the eligibility criteria; 2) missed, if they are presumed eligible but missed due to error or staff availability; or 3) included (eligible and randomised). Study personnel obtain written informed consent from all eligible patients.

In all patients, fractures are initially reduced, followed by plaster cast stabilization or - if this does not sufficiently reduce the fracture - by external fixator. The surgeon on charge with proven expertise in reduction and casting maneuvers performs the reduction and casting. Patients are admitted to the trauma ward afterwards in order to elevate the leg to reduce the swelling around the ankle. Operation will be performed when swelling is reduced to an acceptable level. The patient’s skin is assessed preoperatively ensuring adequate skin wrinkling.

#### Randomisation

Sequentially numbered, opaque, sealed envelopes are used for randomisation. Patients are randomised either to intervention group for arthroscopically assisted open reduction and internal fixation (AORIF) or to control group treated with open reduction and internal fixation (ORIF).

### Intervention

#### Operative technique

Patients in the intervention group are treated by AORIF, patients in the control group by ORIF. The operative treatment is performed by one of the foot and ankle surgeons. Slight differences in the operative procedure depend on fracture type and localisation. The standard operative protocol is the same in all patients no matter whether they are included in the study or not. It will be described in detail in the following.

#### Positioning

The positioning on the operating table depends on whether the posterior malleolus is addressed or not. If the posterior malleolus is not affected the patient is placed in supine position. If the posterior malleolus is addressed the patient is placed on the contralateral side in a so-called unstable lateral position. “Unstable” means that the patient is placed on the contralateral side, but 1) the leg is mobile so that ankle arthroscopy can easily be performed and 2) the patient can simply be turned into supine position after fixation of the posterior and lateral malleolus without changing the sterile cover.

#### Ankle arthroscopy

The arthroscopy is performed first. At this time the fractures are not fixed. Therefore, the joint is unstable and the entire tibiotalar joint can be evaluated without additional distraction. To avoid lesions of the cartilage and soft tissue, the joint is first inflated with saline, and the portals are created by blunt dissection. A 2.7 mm, 30° arthroscope is inserted into the ankle through a standard anteromedial portal. Afterwards the standard anterolateral portal is performed in the same way. A standardized systematic examination as described by Ferkel and Fasulo is regularly used to inspect the internal structures [18]. At this stage all loose bodies and disrupted ligaments extending into the joint are removed. If there are loose fragments that are big enough to be reduced and fixed, resorbable ethipins (Ethicon, Somerville, New Jersey, USA) are used. Chondral damage, if present, is treated according to the grade of damage. In the AORIF group a final examination is performed by ankle arthroscopy after fracture fixation. In this case, we use an elevatorium in order to assess the posterior aspects. Thereby the fracture reduction can be evaluated and all remaining loose bodies can be removed.

#### Classification and treatment of cartilage lesions

Lesions of the articular cartilage are graded according to depth (superficial, partial-thickness, full-thickness cartilage defect) and localization as determined by inspection and probing. The classification system postulated by ICRS (International Cartilage Repair Society) focussing on the lesion depth (grade 0–4) is applied [19]. Therapy depends on the grade of the cartilage lesion. Debridement of unstable edges is suggested for ICRS 2 lesions, whereas ICRS 3 and 4 lesions need further treatment. In these cases, chondroplasty and micro fracturing is applied.

### Fracture fixation

#### Posterior malleolus

**Fig. 1:**
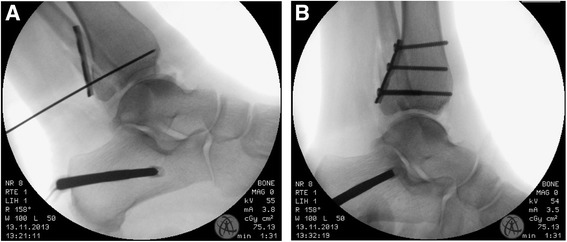
Intraoperative x-ray; **a** Lateral view with temporary k-wire fixation of the posterior fragment; **b** Lateral view after fixation with a three-hole one-third tubular plate for fixation of a posterior malleolus fracture

Until now, there are no clear guidelines for the treatment of posterior malleolus fractures. Historically, the size of the fragment was the major determining factor [20, 21]. When evaluating the available literature, there is no hard evidence for these recommendations as they are only based on a small case series of eight patients by Nelson and Jensen in 1940 [22]. There is growing evidence, that there are more factors that have to be considered when determining appropriate management of posterior malleolus fractures – such as articular impaction, comminution, ankle congruity and syndesmotic stability. Ogilvie-Harris et al. could demonstrate that the posterior inferior tibio fibular ligament (PITFL) provides the major part of syndesmotic stability. This contribution to syndesmotic stability is disrupted when the posterior malleolus is fractured [23]. Principally, the syndesmotic stability can be restored by tibio fibular fixation or ORIF in case of a posterior malleolus fracture. Regarding transsyndesmotic screw fixation, Gardner et al. reported a syndesmotic malreduction rate after screw fixation of 52 % on postoperative CT evaluation [24]. They concluded that anatomic reduction and fixation of a posterior malleolus fracture is superior in re-establishing syndesmotic stability by restoring the length of the intact PITFL. Furthermore, anatomic reduction of the posterior malleolus fragment restores the relationship between the tibia and fibula and facilitates bone-to-bone healing [25]. Based on these considerations, we routinely perform open reduction and internal fixation of posterior malleolus fracture if the size of the fragment allows for it. We fix the posterior malleolus before addressing the fibula to allow adequate visualization of the reduction during x-ray imaging as the fibular plate often interferes with the fracture line on the lateral view [26]. We perform a posterolateral approach [27]. A longitudinal skin incision posterior to the peroneal tendons is performed. Retraction of the peroneal muscles and tendons ventrally visualizes the posterolateral edge of the distal tibia. Care is taken to preserve the PITFL attachment to the fragment and the joint capsule. In the next step, open reduction is performed using K-Wires to fix the anatomic reduction temporary. If the reduction is sufficient a titanium one-third tubular plate with two to four screws is used in an antiglide-technique to perform internal fixation (Fig. 1). If the fracture is simple with two fragments only and the fragment size is big enough two single lag screws are used.

#### Lateral malleolus

**Fig. 2:**
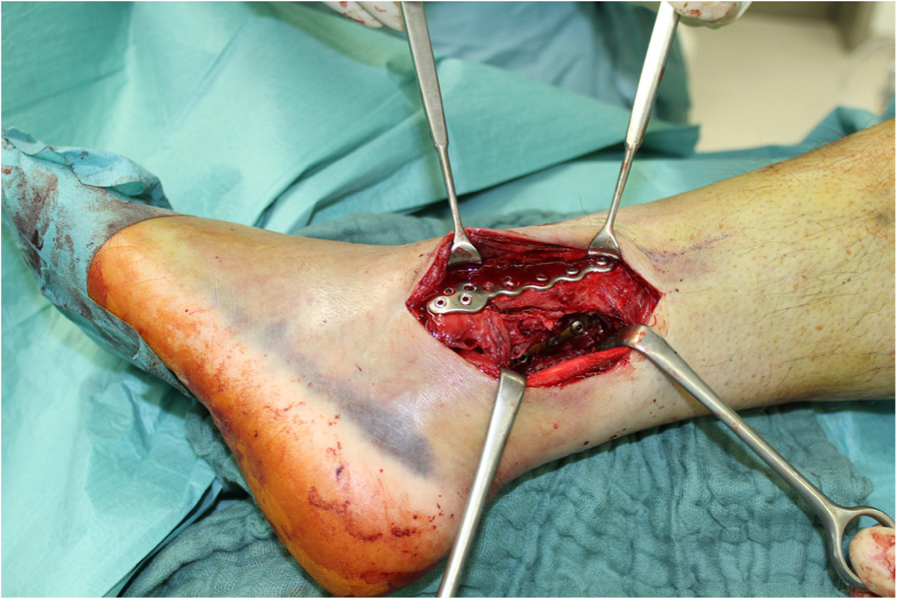
The intraoperative image shows the posterolateral incision and a locking plate used for the lateral malleolus fracture (Arthrex, München, Germany) and one-third tubular plate used for the posterior malleolus fracture (Synthes, Umkirch, Germany)

**Fig. 3:**
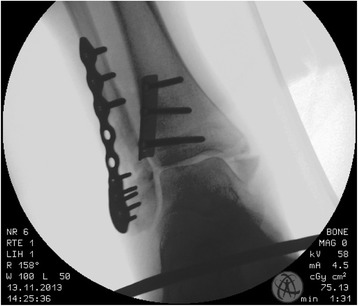
Intraoperative X-ray; Mortise view after fixation of the posterior malleolus with a with an three-hole antiglide-plate and a locking plate for the fracture of the lateral malleolus

If the patients suffer both, a fracture of the posterior and the lateral malleolus, both fractures can be stabilized using the same posterolateral approach. After fixation of the posterior malleolus as described above the preparation of the fibula fracture is performed. Therefore, we prepare a full-thickness-flap in the direction of the fibular superficial to the peroneal tendons (Fig. 2). According to fracture type and localization, we normally use a lag screw performed as it is described in the AO manual followed by a one-third tubular plate as a neutralization plate (Synthes, Umkirch, Germany). In case of a multifragmentary fracture, distal fracture or in case of osteoporotic bone a lateral locking plate or a locking hook-plate is used (Arthrex, München, Germany) (Fig. 3). If the patient only presents with a lateral malleolus fracture, a standard lateral incision is performed.

#### Medial malleolus

**Fig. 4:**
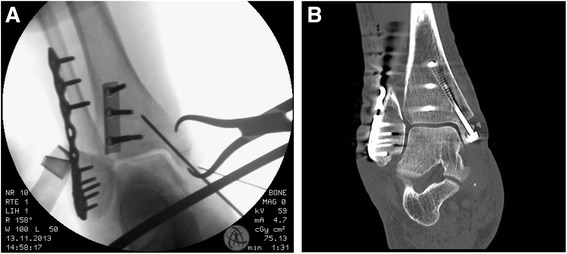
Intraoperative x-ray; **a** Mortise view with an antiglide-plate for the posterior malleolus, locking plate for the fracture of the lateral malleolus and additional temporary k-wire fixation of the medial malleolus. **b** postoperative CT-scan (coronar view) with an antiglide-plate for the posterior malleolus, locking plate for the fracture of the lateral malleolus and lag screw fixation of the medial malleolus

Routinely, an incision beginning 1–2 cm distally to the tip of the medial malleolus over the medial malleolus in the direction of the middle of the distal tibia is performed. In the next step, we start with the exposure of the anterior-medial part of the fracture site with a limited arthrotomy to assure an anatomic reduction at the joint surface. K-wires are inserted perpendicular to the fracture plane and replaced by two cannulated leg screws after the anatomical reduction was proofed (Fig. 4). Washers are used in case of osteoporotic bone. If the fracture is comminuted, the fragment is very distal or small or the bone is osteoporotic, either a locking hook plate or medial tension band wiring is used.

#### Syndesmotic complex

After all fractures have been stabilized, the stability of the syndesmotic complex is tested. Therefore, the “external rotation test” as described by Frick et al. is used [28]. This test is commonly used for the testing of syndesmotic stability. In case of persisting instability, the reduction of the distal tibiofibular joint is performed using a reduction clamp. The reduction is checked by an intraoperative CT-scan (Iso-C-3D, Ziehm Imaging, Nürnberg, Germany). If the reduction is sufficient, we position a TightRope® (Arthrex, München, Germany) 30° from posterior to anterior parallel to the tibial plafond with the ankle in neutral position.

#### Postoperative treatment

The postoperative protocol is the same for all fracture types included. All patients are treated following the same postoperative protocol – independent of study participation and allocation to the two different groups. Postoperatively, the ankle is routinely immobilised in a cast for 1 week. Directed-active and active-assisted range of motion exercises is performed immediately. Partial weight bearing (20 kg) is allowed for 6 weeks postoperatively followed by progressive weight bearing to tolerance.

### Study follow-up

#### Schedule of events and assessments

A subjective evaluation (questionnaires), physical examination and radiographic analysis are performed preoperatively and again 6 weeks, 6 and 12 months postoperatively. The further follow-up visits include yearly examination, as the long-term follow-up is essential regarding the outcome parameter of this trial (Schedule of events see Table 2). Patients enrolled in our study do not need any additional outpatient visits or radiographic investigations. The data that we obtain from the medical records include the patient’s age and sex, date of injury, date of surgery, classification of the ankle fracture, grade and locations of intra-articular lesions such as chondral lesions, loose bodies, ligamentous damage and presence or absence of concurrent synovitis, course of wound healing, pre- and posttraumatic activity level, time to return to work and sports and quality of life. Pseudonymization is performed for all patients’ details.

**Table 2:** Schedule of events

Assessment	Screening	Enrolment	Surgery	6 weeks	6 months	12 months	Yearly follow-up
Screening form	x						
Informed consent	x						
Randomisation		x					
Baseline characteristics form		x					
Ankle characteristics/ Physical examination		x		x	x	x	x
Surgical form			x				
Arthroscopic findings form			x				
Perioperative form			x				
Follow-up form				x	x	x	x
X-ray/CT	x		x	x	(x)	x	(x)
Pain Visual Analogue Scale		x		x	x	x	x
Short-form 12		x		x	x	x	x
American Orthopaedic Foot and Ankle Society Score				x	x	x	x
Japanese Society of Surgery of the Foot Score				x	x	x	x
Olerud and Molander Score				x	x	x	x
Karlsson Score				x	x	x	x
Tegner Activity Scale		x		x	x	x	x
Complications			x	x	x	x	x

#### Subjective outcome assessment

In order to blind subjective evaluation, questionnaires are answered before physical examination is performed. Patients will answer questions about their general satisfaction, pain level (VAS), activity level and any complaints. The Short Form 12 (SF-12) [29] and the Tegner Activity Scale (TAS) [30] are filled in pre- and postoperatively.

#### Physical examination

For the physical examination blinding of the patients is not feasible as the arthroscopy portals are visible. The physical examination consists of the inspection of the hind foot including hind foot axis, ankle swelling and wound healing. The important anatomical landmarks are examined for any painful structure. Clinical tests involve medial and lateral ligament stability and non-weight bearing, assisted dorsal and plantar range of motion measured with a goniometer. The physical examination further includes functional outcome scores. The AOFAS Score (American Orthopaedic Foot and Ankle Society) [31], JSSF Score (Japanese Society of Surgery of the Foot) [32], OMAS (Olerud and Molander Score) [33], Karlsson Score are used to evaluate postoperative functional outcome.

#### Primary outcome

The AOFAS score is the primary outcome parameter. It is one of the most widely used scoring systems to evaluate functional ability and physical examination incorporated in a numeric scale. It has been widely adopted and has become an accepted standard of assessing patients after foot and ankle surgery. A validated German language version is used.

#### Secondary outcome

The JSSF, OMAS, Karlsson Score, TAS and SF-12 are recorded postoperatively. Further secondary outcome parameter are radiographic analysis, arthroscopic findings of intra-articular lesions, time to return to work/sports and complications.

#### Radiographic analysis

The radiographic assessments comprise the routine x-rays (non-weight bearing, a.p. and lateral) and a CT scan preoperatively and postoperatively. Postoperative radiographs and x-rays performed 6 weeks after the operation are non-weight bearing films. Beginning with 1-year of follow-up, weight-bearing films will be obtained. Position of implants, potential secondary dislocation and fracture consolidation/healing are evaluated and documented via radiographic analysis. The displacement of the fracture site is controlled fluoroscopically intra- and postoperatively; displacement is graded into three groups (no dislocation, dislocation ≤2 mm and dislocation >2 mm). In case sub-optimal reduction or malreduction ≥2 mm is recognized using intraoperative x-ray or arthroscopy, fixation will be removed and the reduction will be performed again. Thereafter the reduction is re-evaluated using x-ray and arthroscopy. If malreduction of ≥2 mm should be recognized in postoperative CT-scans revision surgery would be discussed with the patient. Radiographic assessment of posttraumatic osteoarthritis is performed according to the classification system by Kellgren et al. [34].

#### Fracture classification

Detailed description and classification of the fracture are performed using the AO classification system.

#### Arthroscopic findings

**Fig. 5:**
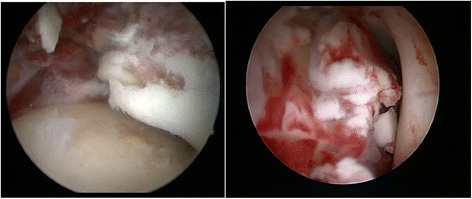
Exemplary arthroscopy images showing an osteochondral lesions grade 4 (ICRS) on the medial talus and loose bodies

The appearance, description and classification of intra-articular findings in the arthroscopic group are documented. Detailed description of the lesion, capsular, ligaments, ventral syndesmosis, grading and localization of chondral lesions (ICRS classification), loose bodies is recorded and analysed. These intraoperative findings are further analysed according to fracture classification. In order to determine the frequency and distribution of the chondral lesions in the series, fractures of the same type will be grouped together and statistical analysis will be performed to compare these groups. In Fig. 5 two examples of intra-articular pathologies are presented.

#### Data and safety monitoring board

According to the Data and Safety Monitoring Board Guidelines (National Institute of Health, NIH) we establish a group of experts (experienced trauma surgeons, who are not involved in our study) who independently review and evaluate the accumulated study data for participant safety, study conduct and progress and also efficiency of our study. In detail, arthroscopical findings and arthroscopy-associated complications will be evaluated thoroughly after 15 patients in the intervention group (AORIF). Based on these results, they will make recommendations concerning continuation, modification or termination of the trial.

### Statistical analysis

Primary outcome of our study is the difference of the AOFAS score between the intervention (AORIF) and comparison (ORIF) group after a follow-up of 2 years. We expect a mean difference of four points between the AORIF and ORIF group showing better results in the AORIF group. This expectation is feasible on the basis of the existing data (see [3] and [1]). The statistical calculation was performed with an expected correlation of 0.5 between the two groups. Based on a conservative estimation of the observed standard deviation of the AOFAS score in a comparable population (see [3] and [1]), we expect a standard deviation of six for the AOFAS score in our population. Based on the supposed correlation of 0.5 the standard deviation of the differences regarding our primary outcome is also expected to be not more than six. Therefore, each group has to contain 37 patients in order to find a difference of four between the two groups with a level of significance of 5 % and a power of 80 % and a standard deviation of six. In case of subgroup analysis, Shapiro-Wilk and Levene tests will be used to test normality and equality of variances. Subgroup analysis will be conducted using Student’s t-test, Pearson Chi-Square or ANOVA where appropriate. Statistics will be calculated using SPSS 22.0 (SPSS Inc. Chicago, Illinois 60606).

## Discussion

The subjective and functional outcome of operatively treated ankle fractures is often poor [5, 6]. Winters et al. reported moderate to poor results in more than 20 % of their patients in simple, isolated fractures of the distal fibula [35]. Furthermore, Day et al. demonstrated in their long-term study, that only 52 % of the patients had good to excellent results following a bimalleolar fracture whereas 48 % of these patients only had a moderate or poor outcome after 10 years [36]. Hong et al. showed that more than 50 % of the patients with bi- or trimalleolar fractures suffered from stiffness and pain after 2 years. In the same collective 18 % did not return to sports after this period of time [37]. Recently, several determining factors for these unsatisfying results have been proposed including age, sex, severity of the fracture, size of the posterior fragment, involvement of the medial malleolus and the quality of anatomic reduction [36, 38–42]. The prerequisite for a satisfactory outcome is the anatomic reduction – at the same time it is the only risk factor that can surgically be influenced. But even anatomic restoration cannot surely prevent poor postoperative results. Based on these considerations, the unrecognized articular injuries involving cartilage and soft tissue might be the reason for these adverse clinical results [2, 8, 9]. Only since arthroscopic studies in ankle fractures exist, the incidence of chondral lesions has become evident. Chondral lesions occur in 20–89 % [3, 15]. A recent review reported chondral lesions in 495 of 782 patients (63,3 %) with more than a half of these lesions localized on the talus [43]. For instance, Hintermann et al. reported 79 % osteochondral lesion (OCL) in acute ankle fractures [5]. Leonartidis et al. found comparable rate of OCLs in their retrospective case series of 83 patients [44]. In their prospective study Stufkens et al. could demonstrate that the traumatic cartilage injury was an independent predictor for the development of posttraumatic osteoarthritis. Further they could show that chondral lesions on the lateral talus and medial malleolus were associated with worse clinical outcome [45]. Until today, there are only two randomised controlled trails evaluating the effect of additional ankle arthroscopy in ankle fractures. In contrast to our study, both studies available only included patients with isolated fractures of the distal fibula at the level of the syndesmosis (AO type 44 B1). These are the most simple fractures that are regularly treated operatively. Thodarson and co-workers found in their very small cohort that eight of nine patients had articular damage to the talar dome in the arthroscopy group [3]. They found better results in favour of AORIF although not significant after 21 months of follow-up [3]. Takao et al. enrolled 72 patients in total with 36 for each group with a follow-up of 3.5 years on average. They documented an osteochondral lesion in 74 % in the AORIF group. Furthermore, the mean AOFAS score was significantly better when patients were treated arthroscopically although the mean difference in AOFAS score was only 3.4 points [1].

Although arthroscopy is increasingly used in the setting of trauma for articular and periarticular fractures of the lower limb, the effectiveness of AORIF compared with ORIF has yet to be determined [46]. To date, currently available literature has not sufficiently proven that treatment of these intra-articular injuries improves outcomes over standard ORIF, as there is only few randomized controlled studies available comparing these two operative techniques. This also applies for fractures to the ankle joint - especially in case of complex fracture patterns - although the intra-articular damage is reported to be even more pronounced the more complex the fracture is [44].

Consequently, we perform the first RCT comparing AORIF to ORIF in complex ankle fractures expecting an even more distinctive effect of arthroscopy in these fracture types. By reporting the results of this first randomised controlled trail, we intend to document on the effectiveness of supplementary ankle arthroscopy in complex fractures to the ankle. By arthroscopically assisted fracture treatment we hope to improve the postoperative subjective and functional results. We believe this is of great importance as these injuries are frequent and debilitating. The main goal of our study is to identify the fracture types that will benefit from additional arthroscopy most.

## Ethics and consent to participate

This study is conducted according to the principles of the Declaration of Helsinki (Version 9, 10/08, Seoul) and in accordance with the Medical Research Involving Human Subjects Act. The Ethical Committee of the Medical Faculty approved the study at the Department of Trauma Surgery of Munich University Hospital (AZ 500-15). All patients included in our study provided written informed consent to participate.

## Consent for publication

Not applicable.

## Availability of data and material

As this is a study protocol, no data are presented.

## Abbreviations

AOFAS Score: American Orthopaedic Foot and Ankle Society
AORIF: arthroscopically assisted open reduction and internal fixation
ICRS: International Cartilage Repair Society
JSSF Score: Japanese Society of Surgery of the Foot
NIH: National Institute of Health
OCL: osteochondral lesion
OMAS: olerud and molasnder score
ORIF: open reduction and internal fixation
PITFL: posterior inferior tibio fibular ligament
RCT: randomised controlled trial
SF-12: a 12-item short-form health survey
TAS: tegner activity scale
VAS: visual analogue scale
